# *cyclo*-Tris[μ-5-(2-pyrid­yl)pyrazol-1-ido-κ^3^ *N* ^1^,*N* ^5^:*N* ^2^]tris­ilver(I)

**DOI:** 10.1107/S1600536809040082

**Published:** 2009-10-10

**Authors:** Zhe An, Ru-Jin Zhou

**Affiliations:** a School of Chemistry and Life Science, Maoming University, Maoming 525000, People’s Republic of China

## Abstract

In the title compound, [Ag_3_(C_8_H_6_N_3_)_3_], the asymmetric unit contains three silver cations and three depronated 5-(2-pyrid­yl)pyrazol-1-ide ligands. Each silver cation is chelated by one 5-(2-pyrid­yl)pyrazol-1-ide ligand, which also acts as a bridging ligand towards the next silver ion *via* the second pyrazole N atom. In summary, three silver cations and three deprotonated 3-(2-pyrid­yl)-1*H*-pyrazole ligands produce a macrocyclic trimeric coordination oligomer that exhibits an almost planar conformation (mean deviation 0.1483 Å). In addition, short non-bonding Ag⋯Ag inter­actions [3.127 (2) Å] are observed.

## Related literature

For coordination compounds with pyridyl-pyrazolide ligands, see: Ward *et al.* (1998 ▶, 2001 ▶).
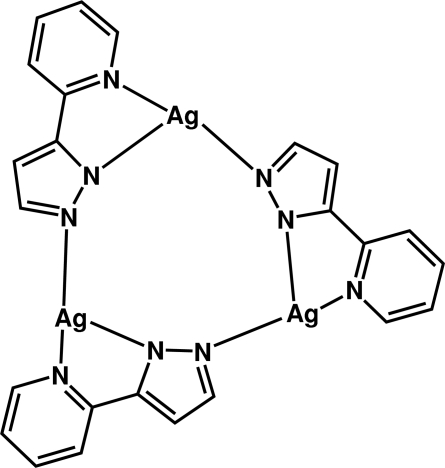


## Experimental

### 

#### Crystal data

[Ag_3_(C_8_H_6_N_3_)_3_]
*M* *_r_* = 756.07
Monoclinic, 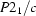

*a* = 11.597 (9) Å
*b* = 8.555 (6) Å
*c* = 25.52 (2) Å
β = 103.018 (9)°
*V* = 2467 (3) Å^3^
*Z* = 4
Mo *K*α radiation
μ = 2.39 mm^−1^
*T* = 294 K
0.10 × 0.10 × 0.08 mm

#### Data collection

Bruker SMART CCD diffractometer
Absorption correction: multi-scan (*SADABS*; Bruker, 2001 ▶) *T*_min_ = 0.796, *T*_max_ = 0.832
12411 measured reflections
4294 independent reflections
2947 reflections with *I* > 2σ(*I*)
*R*_int_ = 0.041

#### Refinement

*R*[*F* ^2^ > 2σ(*F* ^2^)] = 0.033
*wR*(*F* ^2^) = 0.064
*S* = 1.00
4294 reflections
325 parameters
H-atom parameters not refined
Δρ_max_ = 0.41 e Å^−3^
Δρ_min_ = −0.41 e Å^−3^

### 

Data collection: *SMART* (Bruker, 2002 ▶); cell refinement: *SAINT-Plus* (Bruker, 2003 ▶); data reduction: *SAINT-Plus*; program(s) used to solve structure: *SHELXTL* (Sheldrick, 2008 ▶); program(s) used to refine structure: *SHELXL97* (Sheldrick, 2008 ▶); molecular graphics: *SHELXTL*; software used to prepare material for publication: *SHELXTL*.

## Supplementary Material

Crystal structure: contains datablocks I, global. DOI: 10.1107/S1600536809040082/im2146sup1.cif

Structure factors: contains datablocks I. DOI: 10.1107/S1600536809040082/im2146Isup2.hkl

Additional supplementary materials:  crystallographic information; 3D view; checkCIF report

## Figures and Tables

**Fig. 1.**
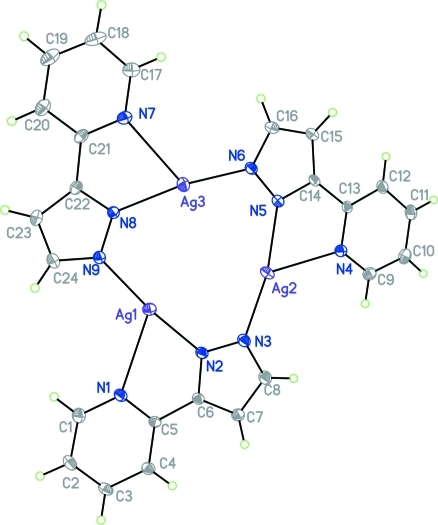
A view of the title compound with the atom-labelling scheme. Displacement ellipsoids are drawn at the 30% probability level.

## supplementary crystallographic information

### Comment

Deprotonated 3-(2-pyridyl)pyrazole is a potentially tridentate ligands and it's derivatives have been widely used for the construction of supramolecular architectures by their ability of producing coordination compounds (Ward *et al.* 1998; 2001). As a continuation of these studies, we now report the crystal structure of the title complex.

As shown in figure 1, the asymmetric unit contains three silver cations and three depronated 5-(2-pyridyl)pyrazol-1-ide ligands. Each silver cation is chelated with one 5-(2-pyridyl)pyrazol-1-ide ligand, which in addition acts as a bridging ligand towards the next silver ion *via* the second pyrazole nitrogen.

In summary, three silver cations and three depronated 5-(2-pyridyl)pyrazol-1-ide ligands produce a macrocyclic trimeric coordination oligomer that exhibits an almost planar conformation. In addition, short non-bonding Ag-Ag interactions (3.127 (2) Å) are observed.

### Experimental

The synthesis of the title compound is performed in 25 ml Teflon-lined stainless steel vessels. Powdered 3-(2-pyridyl)pyrazole (1 mmol) together with silver nitrate (1 mmol) is heated to 170°C in 10 ml of a water/ethanol mixture (1:1) for 24 h. Colorless crystals were obtained after cooling to room temperature.

### Refinement

All hydrogen atoms were positioned geometrically and were refined using a riding model with C—H = 0.93 Å and *U*_iso_(H) = 1.2Ueq(C).

### Crystal data

**Table:**

[Ag_3_(C_8_H_6_N_3_)_3_]	*F*(000) = 1464
*M**_r_* = 756.07	*D*_x_ = 2.036 Mg m^−^^3^
Monoclinic, *P*2_1_/*c*	Mo *K*α radiation, λ = 0.71073 Å
Hall symbol: -P 2ybc	Cell parameters from 843 reflections
*a* = 11.597 (9) Å	θ = 2.9–20.3°
*b* = 8.555 (6) Å	µ = 2.39 mm^−^^1^
*c* = 25.52 (2) Å	*T* = 294 K
β = 103.018 (9)°	Block, colorless
*V* = 2467 (3) Å^3^	0.10 × 0.10 × 0.08 mm
*Z* = 4	

### Data collection

**Table:**

Bruker SMART CCD diffractometer	4294 independent reflections
Radiation source: fine-focus sealed tube	2947 reflections with *I* > 2σ(*I*)
graphite	*R*_int_ = 0.041
φ and ω scans	θ_max_ = 25.0°, θ_min_ = 1.6°
Absorption correction: multi-scan (*SADABS*; Bruker, 2001)	*h* = −13→13
*T*_min_ = 0.796, *T*_max_ = 0.832	*k* = −9→10
12411 measured reflections	*l* = −30→27

### Refinement

**Table:**

Refinement on *F*^2^	Primary atom site location: structure-invariant direct methods
Least-squares matrix: full	Secondary atom site location: difference Fourier map
*R*[*F*^2^ > 2σ(*F*^2^)] = 0.033	Hydrogen site location: inferred from neighbouring sites
*wR*(*F*^2^) = 0.064	H-atom parameters not refined
*S* = 1.00	*w* = 1/[σ^2^(*F*_o_^2^) + (0.02*P*)^2^] where *P* = (*F*_o_^2^ + 2*F*_c_^2^)/3
4294 reflections	(Δ/σ)_max_ = 0.005
325 parameters	Δρ_max_ = 0.41 e Å^−^^3^
0 restraints	Δρ_min_ = −0.41 e Å^−^^3^

### Special details

**Table:**

Geometry. All e.s.d.'s (except the e.s.d. in the dihedral angle between two l.s. planes) are estimated using the full covariance matrix. The cell e.s.d.'s are taken into account individually in the estimation of e.s.d.'s in distances, angles and torsion angles; correlations between e.s.d.'s in cell parameters are only used when they are defined by crystal symmetry. An approximate (isotropic) treatment of cell e.s.d.'s is used for estimating e.s.d.'s involving l.s. planes.
Refinement. Refinement of *F*^2^ against ALL reflections. The weighted *R*-factor *wR* and goodness of fit *S* are based on *F*^2^, conventional *R*-factors *R* are based on *F*, with *F* set to zero for negative *F*^2^. The threshold expression of *F*^2^ > σ(*F*^2^) is used only for calculating *R*-factors(gt) *etc*. and is not relevant to the choice of reflections for refinement. *R*-factors based on *F*^2^ are statistically about twice as large as those based on *F*, and *R*- factors based on ALL data will be even larger.

### Fractional atomic coordinates and isotropic or equivalent isotropic displacement parameters (Å^2^)

**Table:**

	*x*	*y*	*z*	*U*_iso_*/*U*_eq_	
Ag1	0.22973 (3)	0.98307 (4)	0.144566 (15)	0.06499 (13)	
Ag2	0.13700 (3)	0.74112 (4)	0.019289 (14)	0.06424 (13)	
C1	0.4980 (4)	1.0995 (6)	0.2419 (2)	0.0747 (15)	
H1	0.4483	1.1577	0.2584	0.090*	
C2	0.6185 (4)	1.1006 (6)	0.26491 (19)	0.0719 (14)	
H2	0.6490	1.1581	0.2959	0.086*	
C3	0.6909 (4)	1.0148 (5)	0.24072 (19)	0.0659 (14)	
H3	0.7720	1.0113	0.2553	0.079*	
C4	0.6424 (4)	0.9331 (5)	0.19436 (18)	0.0580 (12)	
H4	0.6911	0.8759	0.1771	0.070*	
C5	0.5212 (4)	0.9362 (5)	0.17348 (17)	0.0470 (11)	
C6	0.4645 (4)	0.8456 (5)	0.12563 (17)	0.0504 (11)	
C7	0.5111 (4)	0.7483 (6)	0.09242 (19)	0.0708 (14)	
H7	0.5904	0.7248	0.0949	0.085*	
C8	0.4147 (4)	0.6935 (6)	0.0548 (2)	0.0727 (15)	
H8	0.4187	0.6258	0.0268	0.087*	
C9	0.1397 (4)	0.4507 (6)	−0.0809 (2)	0.0657 (13)	
H9	0.2208	0.4457	−0.0667	0.079*	
C10	0.0916 (5)	0.3499 (6)	−0.1224 (2)	0.0731 (14)	
H10	0.1390	0.2807	−0.1362	0.088*	
C11	−0.0290 (5)	0.3556 (6)	−0.14265 (19)	0.0741 (15)	
H11	−0.0648	0.2889	−0.1703	0.089*	
C12	−0.0958 (4)	0.4609 (5)	−0.12148 (18)	0.0635 (13)	
H12	−0.1774	0.4655	−0.1344	0.076*	
C13	−0.0391 (4)	0.5615 (5)	−0.08009 (16)	0.0476 (11)	
C14	−0.1064 (4)	0.6755 (5)	−0.05658 (16)	0.0484 (11)	
C15	−0.2255 (4)	0.7133 (6)	−0.06914 (19)	0.0658 (14)	
H15	−0.2846	0.6698	−0.0959	0.079*	
C16	−0.2375 (4)	0.8301 (6)	−0.03316 (19)	0.0653 (14)	
H16	−0.3081	0.8796	−0.0318	0.078*	
N1	0.4493 (3)	1.0192 (4)	0.19734 (15)	0.0632 (11)	
N2	0.3451 (3)	0.8464 (4)	0.10893 (14)	0.0544 (10)	
N3	0.3148 (3)	0.7521 (4)	0.06469 (15)	0.0619 (10)	
N4	0.0777 (3)	0.5539 (4)	−0.05982 (14)	0.0555 (10)	
N5	−0.0503 (3)	0.7659 (4)	−0.01505 (14)	0.0518 (9)	
N6	−0.1319 (3)	0.8612 (4)	−0.00053 (14)	0.0552 (10)	
Ag3	−0.08697 (3)	1.00054 (4)	0.069660 (14)	0.06312 (13)	
N7	−0.2477 (3)	1.1562 (4)	0.10333 (16)	0.0636 (11)	
N8	−0.0152 (3)	1.0984 (4)	0.14786 (13)	0.0522 (9)	
C17	−0.3626 (5)	1.1848 (6)	0.0800 (2)	0.0807 (16)	
H17	−0.3968	1.1320	0.0484	0.097*	
C21	−0.1995 (4)	1.2328 (5)	0.1486 (2)	0.0568 (12)	
C22	−0.0746 (4)	1.1992 (5)	0.17237 (18)	0.0531 (12)	
N9	0.0977 (3)	1.0881 (4)	0.17771 (14)	0.0565 (10)	
C18	−0.4303 (5)	1.2879 (8)	0.1008 (3)	0.101 (2)	
H18	−0.5090	1.3046	0.0836	0.122*	
C20	−0.2641 (5)	1.3382 (6)	0.1718 (2)	0.0766 (15)	
H20	−0.2292	1.3894	0.2035	0.092*	
C23	0.0005 (4)	1.2554 (5)	0.21894 (19)	0.0638 (13)	
H23	−0.0174	1.3265	0.2435	0.077*	
C24	0.1063 (4)	1.1833 (6)	0.22055 (19)	0.0667 (13)	
H24	0.1745	1.1978	0.2474	0.080*	
C19	−0.3813 (6)	1.3662 (7)	0.1470 (3)	0.099 (2)	
H19	−0.4260	1.4372	0.1617	0.118*	

### Atomic displacement parameters (Å^2^)

**Table:**

	*U*^11^	*U*^22^	*U*^33^	*U*^12^	*U*^13^	*U*^23^
Ag1	0.0523 (2)	0.0727 (3)	0.0678 (3)	0.01114 (19)	0.00901 (18)	−0.0029 (2)
Ag2	0.0542 (2)	0.0702 (3)	0.0589 (2)	−0.00637 (19)	−0.00713 (17)	0.0037 (2)
C1	0.076 (4)	0.073 (4)	0.075 (4)	0.002 (3)	0.016 (3)	−0.020 (3)
C2	0.072 (4)	0.073 (4)	0.060 (3)	−0.002 (3)	−0.009 (3)	−0.011 (3)
C3	0.051 (3)	0.066 (3)	0.069 (4)	0.001 (3)	−0.010 (3)	0.000 (3)
C4	0.052 (3)	0.059 (3)	0.059 (3)	0.005 (2)	0.005 (2)	0.003 (3)
C5	0.043 (3)	0.046 (3)	0.049 (3)	0.004 (2)	0.004 (2)	0.006 (2)
C6	0.046 (3)	0.052 (3)	0.049 (3)	0.009 (2)	0.001 (2)	0.002 (2)
C7	0.052 (3)	0.085 (4)	0.072 (3)	0.018 (3)	0.007 (3)	−0.017 (3)
C8	0.068 (3)	0.076 (4)	0.069 (4)	0.014 (3)	0.005 (3)	−0.019 (3)
C9	0.064 (3)	0.065 (4)	0.071 (4)	−0.001 (3)	0.022 (3)	0.005 (3)
C10	0.094 (4)	0.058 (4)	0.075 (4)	0.004 (3)	0.035 (3)	−0.001 (3)
C11	0.094 (4)	0.068 (4)	0.060 (4)	−0.012 (3)	0.017 (3)	−0.016 (3)
C12	0.067 (3)	0.070 (4)	0.051 (3)	−0.008 (3)	0.007 (3)	−0.009 (3)
C13	0.053 (3)	0.052 (3)	0.038 (3)	−0.007 (2)	0.010 (2)	0.003 (2)
C14	0.047 (3)	0.055 (3)	0.037 (3)	−0.007 (2)	−0.003 (2)	0.005 (2)
C15	0.053 (3)	0.079 (4)	0.059 (3)	−0.003 (3)	−0.001 (2)	−0.007 (3)
C16	0.052 (3)	0.072 (4)	0.066 (4)	0.005 (3)	0.001 (3)	0.004 (3)
N1	0.052 (2)	0.071 (3)	0.060 (3)	0.006 (2)	0.000 (2)	−0.011 (2)
N2	0.046 (2)	0.056 (2)	0.057 (2)	0.0048 (18)	0.0026 (18)	−0.007 (2)
N3	0.059 (2)	0.062 (3)	0.059 (3)	0.008 (2)	0.002 (2)	−0.007 (2)
N4	0.054 (2)	0.056 (3)	0.056 (2)	0.0006 (19)	0.012 (2)	−0.001 (2)
N5	0.052 (2)	0.052 (2)	0.048 (2)	−0.002 (2)	0.0045 (18)	−0.0009 (19)
N6	0.053 (2)	0.059 (3)	0.049 (2)	0.003 (2)	0.0016 (19)	−0.0032 (19)
Ag3	0.0699 (3)	0.0608 (3)	0.0563 (2)	−0.00182 (19)	0.00949 (19)	−0.00739 (19)
N7	0.055 (3)	0.067 (3)	0.071 (3)	0.008 (2)	0.018 (2)	0.006 (2)
N8	0.048 (2)	0.055 (2)	0.052 (2)	0.0061 (18)	0.0074 (19)	−0.0068 (19)
C17	0.066 (4)	0.082 (4)	0.093 (4)	0.004 (3)	0.014 (3)	0.010 (3)
C21	0.061 (3)	0.049 (3)	0.069 (3)	0.001 (2)	0.034 (3)	0.009 (3)
C22	0.061 (3)	0.049 (3)	0.055 (3)	0.001 (2)	0.025 (3)	0.003 (2)
N9	0.054 (2)	0.059 (2)	0.055 (2)	0.0018 (19)	0.009 (2)	−0.007 (2)
C18	0.061 (4)	0.106 (5)	0.144 (7)	0.024 (4)	0.039 (4)	0.022 (5)
C20	0.090 (4)	0.066 (4)	0.082 (4)	0.016 (3)	0.038 (3)	0.007 (3)
C23	0.081 (3)	0.058 (3)	0.058 (3)	0.006 (3)	0.026 (3)	−0.003 (3)
C24	0.074 (4)	0.067 (3)	0.055 (3)	−0.009 (3)	0.006 (3)	−0.007 (3)
C19	0.092 (5)	0.093 (5)	0.125 (6)	0.033 (4)	0.053 (4)	0.016 (4)

### Geometric parameters (Å, °)

**Table:**

Ag1—N9	2.110 (4)	C13—N4	1.338 (5)
Ag1—N2	2.129 (4)	C13—C14	1.460 (6)
Ag1—N1	2.617 (4)	C14—N5	1.354 (5)
Ag2—N3	2.127 (4)	C14—C15	1.384 (6)
Ag2—N5	2.162 (4)	C15—C16	1.385 (6)
Ag2—N4	2.547 (4)	C15—H15	0.9300
Ag2—Ag3^i^	3.1274 (17)	C16—N6	1.343 (5)
C1—N1	1.340 (5)	C16—H16	0.9300
C1—C2	1.389 (6)	N2—N3	1.368 (4)
C1—H1	0.9300	N5—N6	1.361 (4)
C2—C3	1.363 (6)	N6—Ag3	2.117 (4)
C2—H2	0.9300	Ag3—N8	2.151 (4)
C3—C4	1.380 (6)	Ag3—N7	2.590 (4)
C3—H3	0.9300	Ag3—Ag2^i^	3.1274 (17)
C4—C5	1.387 (5)	N7—C21	1.337 (5)
C4—H4	0.9300	N7—C17	1.354 (6)
C5—N1	1.341 (5)	N8—C22	1.343 (5)
C5—C6	1.470 (5)	N8—N9	1.362 (4)
C6—N2	1.354 (5)	C17—C18	1.365 (7)
C6—C7	1.382 (6)	C17—H17	0.9300
C7—C8	1.381 (6)	C21—C20	1.387 (6)
C7—H7	0.9300	C21—C22	1.467 (6)
C8—N3	1.338 (5)	C22—C23	1.391 (6)
C8—H8	0.9300	N9—C24	1.349 (5)
C9—N4	1.326 (5)	C18—C19	1.365 (8)
C9—C10	1.383 (6)	C18—H18	0.9300
C9—H9	0.9300	C20—C19	1.385 (7)
C10—C11	1.378 (6)	C20—H20	0.9300
C10—H10	0.9300	C23—C24	1.365 (6)
C11—C12	1.376 (6)	C23—H23	0.9300
C11—H11	0.9300	C24—H24	0.9300
C12—C13	1.406 (6)	C19—H19	0.9300
C12—H12	0.9300		
			
N9—Ag1—N2	171.09 (14)	C5—N1—C1	118.1 (4)
N9—Ag1—N1	116.95 (14)	C5—N1—Ag1	109.6 (3)
N2—Ag1—N1	69.66 (13)	C1—N1—Ag1	132.3 (3)
N3—Ag2—N5	168.08 (14)	C6—N2—N3	108.1 (3)
N3—Ag2—N4	120.99 (13)	C6—N2—Ag1	124.4 (3)
N5—Ag2—N4	70.93 (13)	N3—N2—Ag1	127.4 (3)
N3—Ag2—Ag3^i^	111.26 (10)	C8—N3—N2	107.7 (4)
N5—Ag2—Ag3^i^	68.36 (9)	C8—N3—Ag2	130.9 (3)
N4—Ag2—Ag3^i^	84.05 (10)	N2—N3—Ag2	120.9 (3)
N1—C1—C2	123.6 (5)	C9—N4—C13	118.0 (4)
N1—C1—H1	118.2	C9—N4—Ag2	131.7 (3)
C2—C1—H1	118.2	C13—N4—Ag2	110.2 (3)
C3—C2—C1	118.0 (4)	C14—N5—N6	108.3 (3)
C3—C2—H2	121.0	C14—N5—Ag2	121.5 (3)
C1—C2—H2	121.0	N6—N5—Ag2	130.1 (3)
C2—C3—C4	119.1 (4)	C16—N6—N5	107.8 (4)
C2—C3—H3	120.4	C16—N6—Ag3	130.7 (3)
C4—C3—H3	120.4	N5—N6—Ag3	120.9 (3)
C3—C4—C5	120.1 (4)	N6—Ag3—N8	166.96 (13)
C3—C4—H4	119.9	N6—Ag3—N7	120.80 (14)
C5—C4—H4	119.9	N8—Ag3—N7	69.45 (14)
N1—C5—C4	121.0 (4)	N6—Ag3—Ag2^i^	79.27 (11)
N1—C5—C6	116.7 (4)	N8—Ag3—Ag2^i^	111.59 (10)
C4—C5—C6	122.3 (4)	N7—Ag3—Ag2^i^	81.24 (9)
N2—C6—C7	108.9 (4)	C21—N7—C17	118.1 (4)
N2—C6—C5	119.5 (4)	C21—N7—Ag3	110.3 (3)
C7—C6—C5	131.5 (4)	C17—N7—Ag3	131.1 (4)
C8—C7—C6	105.3 (4)	C22—N8—N9	108.1 (4)
C8—C7—H7	127.4	C22—N8—Ag3	123.4 (3)
C6—C7—H7	127.4	N9—N8—Ag3	128.0 (3)
N3—C8—C7	110.0 (4)	C18—C17—N7	122.7 (5)
N3—C8—H8	125.0	C18—C17—H17	118.6
C7—C8—H8	125.0	N7—C17—H17	118.6
N4—C9—C10	124.3 (5)	N7—C21—C20	121.7 (5)
N4—C9—H9	117.8	N7—C21—C22	116.3 (4)
C10—C9—H9	117.8	C20—C21—C22	122.0 (5)
C9—C10—C11	117.8 (5)	N8—C22—C23	109.4 (4)
C9—C10—H10	121.1	N8—C22—C21	119.7 (4)
C11—C10—H10	121.1	C23—C22—C21	130.9 (4)
C12—C11—C10	119.3 (5)	C24—N9—N8	107.5 (3)
C12—C11—H11	120.3	C24—N9—Ag1	130.9 (3)
C10—C11—H11	120.3	N8—N9—Ag1	119.8 (3)
C11—C12—C13	119.1 (5)	C17—C18—C19	119.3 (6)
C11—C12—H12	120.5	C17—C18—H18	120.3
C13—C12—H12	120.5	C19—C18—H18	120.4
N4—C13—C12	121.5 (4)	C21—C20—C19	119.2 (5)
N4—C13—C14	117.5 (4)	C21—C20—H20	120.4
C12—C13—C14	121.0 (4)	C19—C20—H20	120.4
N5—C14—C15	109.0 (4)	C24—C23—C22	104.8 (4)
N5—C14—C13	119.8 (4)	C24—C23—H23	127.6
C15—C14—C13	131.2 (4)	C22—C23—H23	127.6
C14—C15—C16	105.0 (4)	N9—C24—C23	110.3 (4)
C14—C15—H15	127.5	N9—C24—H24	124.9
C16—C15—H15	127.5	C23—C24—H24	124.9
N6—C16—C15	109.9 (4)	C18—C19—C20	119.0 (6)
N6—C16—H16	125.1	C18—C19—H19	120.5
C15—C16—H16	125.1	C20—C19—H19	120.5

Symmetry codes: (i) −*x*, −*y*+2, −*z*.
